# Low FAT4 expression is associated with a poor prognosis in gastric cancer patients

**DOI:** 10.18632/oncotarget.23702

**Published:** 2017-12-26

**Authors:** Xiaoting Jiang, Zhengchuang Liu, Yingjie Xia, Jungang Luo, Ji Xu, Xujun He, Houquan Tao

**Affiliations:** ^1^ Key Laboratory of Gastroenterology of Zhejiang Province, Zhejiang Provincial People's Hospital, People's Hospital of Hangzhou Medical College, Hangzhou 310014, Zhejiang, China; ^2^ Department of Surgery, Zhejiang Provincial People's Hospital, People's Hospital of Hangzhou Medical College, Hangzhou 310014, Zhejiang, China

**Keywords:** FAT4, gastric cancer, prognosis, methylation, distant metastasis

## Abstract

In this study, we investigated the role of Fat atypical cadherin 4 (FAT4) in gastric cancer (GC) progression. Immunohistochemical analysis showed lower FAT4 expression in tumor tissues from GC patients than in normal gastric epithelium. Lower FAT4 expression was associated with poor prognosis, tumor size and invasion, and lymph node and distant metastases. Multivariate analysis showed that TNM stage, lymph node and distant metastases, Lauren classification, and FAT4 expression were independent prognostic factors in GC. Methylation-specific PCR analysis showed increased *FAT4* promoter methylation in GC tumor tissues and cell lines. Higher *FAT4* promoter methylation was associated with low FAT4 expression and a poor prognosis. BGC-823 cells showed increased FAT4 expression upon treatment with 5-azacytidine, demethylating agent. *FAT4* knockdown in BGC-823 cells led to increased cell proliferation, migration and invasiveness. Moreover, xenografts of BGC-823 cells with *FAT4* knockdown showed enhanced tumor growth and metastasis in nude mice. These findings demonstrate that low FAT4 expression is associated with a poor prognosis in GC patients.

## INTRODUCTION

Despite recent developments in the surgical techniques and improved efficacy of anticancer drugs, mortality rates of gastric cancer (GC) patients remains very high. The overall 5-year survival rate of GC is about 40% because of late diagnosis (stage III and IV), which is associated with lymph node and distant metastasis [1]. Therefore, new prognostic and therapeutic biomarkers are necessary to improve the survival of GC patients.

The Fat gene family was originally identified in *Drosophila* as a member of the cadherin super-family with tumor suppressor functions [2, 3]. It regulates cell proliferation and planar cell polarity during Drosophila development by the Hippo signaling pathway [4, 5]. They encode a type 1 trans-membrane protein with 34 cadherin repeats, 4 epidermal growth factor (EGF)-like repeats, a transmembrane domain and a cytoplasmic domain that is distinct from the classical cadherin proteins [6, 7]. In humans, four members of the Fat family have been identified, namely, FAT1, FAT2, FAT3 and FAT4, which are structurally similar to the Drosophila Fat protein [3]. In mammals, FAT4 is the true structural ortholog of the Drosophila FAT, whereas FAT1, FAT2 and FAT3 are closely related to the Drosophila Fat-like 8 (Fatl-8). FAT4 mediates key developmental functions such as planar cell polarity and regulates the Hippo signaling pathway, which controls the size of the organs [3, 4].

Whole-exome sequencing revealed that *TP53*, *PIK3CA* and *ARID1A* were critical cancer driving genes in GC and cell adhesion genes were frequently mutated [8]. Genomic mutations (5%) and deletions (4%) in FAT4 were observed in GC, whereas FAT4 silencing decreased cellular adhesion and increased migration and invasion of GC cells [8]. FAT4 suppresses growth and invasion in breast [9] and GC cancers [10-12] by activating the Yes-associated protein (YAP) and/or WNT signaling and silencing the epigenetic modifier proteins. However, the definitive role of FAT4 in GC distant metastases remains unknown. Therefore, in this study, we investigated the role of FAT4 in GC progression and prognosis.

## RESULTS

### FAT4 expression is associated with poor prognosis in GC patients

**Figure 1:**
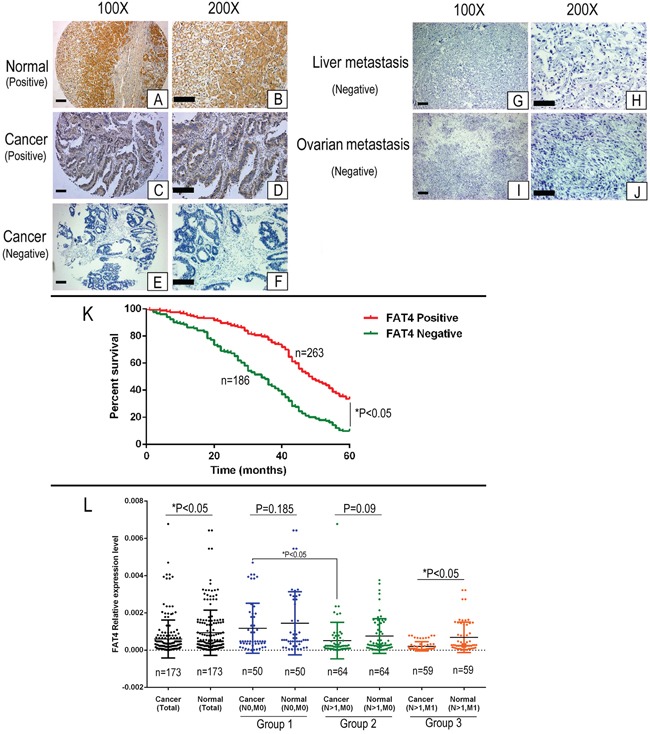
Immunohistochemical analysis of FAT4 in GC patient tissues and Kaplan–Meier survival analysis **(A-B)** Representative IHC images (100X and 200X) showing positive FAT4 expression in normal gastric epithelium. Scale bar, 100 μm. **(C-D)** Representative IHC images (100X and 200X) showing positive FAT4 expression in GC patient tissue. Scale bar, 100 μm. **(E-F)** Representative IHC images (100X and 200X) showing negative FAT4 expression in GC patient tissue. Scale bar, 100 μm. **(G-H)** Representative IHC images (100X and 200X) showing negative FAT4 expression in liver metastatic tissue from GC patients. Scale bar, 100 μm. **(I-J)** Representative IHC images (100X and 200X) showing negative FAT4 expression in ovarian metastatic tissue from GC patients. Scale bar, 100 μm. **(K)** Kaplan–Meier analysis showing survival curves of GC patients (n=449) with high (red) or low/no (green) FAT4 expression Note:^*^ denotes P<0.05 compared to high FAT4 expressing GC patients. **(L)** qRT-PCR analysis of FAT4 mRNA expression in 173 paired GC and adjacent noncancerous patient tissue samples. FAT4 was down-regulated in the tumors than adjacent noncancerous tissues (n=173, *P*<0.05). We classified the patient samples (n=179) based on lymph node and distant metastases status into three groups namely, group1 (N0, M0, n=50), group 2 (N>1, M0, n=64) and group 3 (N>1, M1, n=59). There is no difference of FAT4 expression between cancer tissue and adjacent noncancerous tissues in group1 and group2 (*P*>0.05), but in group3 the FAT4 expression in cancer tissue is significant lower than adjacent noncancerous tissues (*P*<0.05). FAT4 expression was significant lower in cancer tissues from group 2 and group3 patients than group 1 patients (*P*<0.05).

Table 1 shows the association between FAT4 expression and clinicopathological features based on IHC analysis of 449 clinical GC tissue microarray (TMA) samples. FAT4 staining was exclusively cytoplasmic in normal gastric epithelial cells and few cases of tumor cells (Figure 1). Low FAT4 expression was observed in GC tissues than in adjacent noncancerous tissues (Figure 1A-1F). FAT4 expression was detected in 263 (58.6%) out of 449 GC tumor samples analyzed. FAT4 expression reduced from 75.9% to 32.4% in the patients with lymphatic metastasis with increasing N grades (N0 to N4 grades). FAT4 expression decreased from 89.3% to 20.3% with increasing TNM grades (TNM Grade I-IV, *P*<0.05). FAT4 expression was lower in GC patients with lymph node metastasis (48.4%, or 137/283) than patients without lymph node metastasis (75.9%, or 126/166, *P*<0.05). FAT4 expression was lower in patients with distant metastasis (20.3%, or 13/64) than patients without distant metastasis (64.9%, or 250/385; *P*<0.05). Lower FAT4 expression correlated with tumor size, tumor invasion depth (T Grade) and vascular invasion (*P*<0.05, Table 1). FAT4 expression was not associated with age, sex, location, differentiation, Lauren classification and histological type of GC (*P*>0.05, Table 1).

**Table 1:** Association between FAT4 expression and clinicopathological factors

Clinical parameters	FAT4			*P*
	Positive	Negative	t/χ^2^	
Age(yrs)	58.35±12.11	60.72±12.70	1.995	0.872
Gender			0.000	0.996
Male	188(71.5%)	133(28.5%)		
Female	75(71.5%)	53(28.5%)		
Location			5.227	0.073
Proximal	26(44.8%)	32(55.2%)		
Middle	104(61.2%)	66(38.8%)		
Distal	133(60.2%)	88(39.8%)		
Size			15483	0.000
≥5cm	91(47.9%)	99(52.1%)		
<5cm	172(66.4%)	87(33.6%)		
Histology type			1.448	0.694
Papillary adenocarcinoma	9(56.3%)	7(43.7%)		
Tubular adenocarcinoma	195(57.9%)	142(42.1%)		
Mucinous adenocarcinoma	15(53.6%)	13(46.4%)		
Signet-ring cell carcinoma	44(64.7%)	24(35.3%)		
Lauren classification			0.616	0.432
Diffuse type	161(60.1%)	107(39.9%)		
Intestinal type	102(56.4%)	79(43.6%)		
Differentiation			2.075	0.557
Well	9(69.2%)	4(30.8%)		
Moderately	77(58.3%)	55(41.7%)		
Poorly	175(57.9%)	127(42.1%)		
Undifferentiation	2(100%)	0(0.0%)		
Invasion Depth (T Grade)			52.860	0.000
T1	52(89.7%)	6(10.3%)		
T2	84(71.8%)	33(28.2%)		
T3	120(48.4%)	128(51.6%)		
T4	7(36.8%)	19(63.2%)		
Lymphatic Metastasis (N Grade)			53.868	0.000
N0	126(75.9%)	40(24.1%)		
N1	46(66.7%)	23(33.3%)		
N2	57(52.3%)	52(47.7%)		
N3	34(32.4%)	71(67.6%)		
Distant metastasis (M Grade)			45.033	0.000
M0	250(64.9%)	135(35.1%)		
M1	13(19.7%)	51(80.7%)		
TNM Stages			97.426	0.000
I	92(89.3%)	11(10.7%)		
II	107(67.7%)	53(32.3%)		
III	51(41.8%)	71(58.2%)		
IV	13(20.3%)	51(79.7%)		
Lymphatic metastasis			32.594	0.000
Yes	137(48.4%)	146(51.6%)		
No	126(75.9%)	40(24.1%)		
Vascular invasion			29.396	0.000
No	135(73.7%)	48(26.3%)		
Yes	128(48.1%)	138(51.9%)		

All cases were classified according to the World Health Organization's (2010) pathological classification of gastric cancer. Invasion Depth (T Grade) grade T1 includes T1a and T1b, T4 includes T4a and T4b. Lymphatic Metastasis (N Grade) grade N3 includes N3a and N3b. TNM grade I includes Ia and Ib, TNM grade II includes IIa and IIb, TNM grade III includes IIIa, IIIb and IIIc.

### FAT4 expression is associated with GC prognosis

We analyzed if FAT4 expression was associated with GC prognosis. The median survival time was lower for FAT4 negative patients than in FAT4 positive patients (34. 00± 1.84 months *vs.* 49.00 ±1.96 months, P<0.01). The 5-year survival rate of the FAT4 negative patients was lower than that of FAT4 positive patients (9.1% vs. 33.5%, *P*<0.05, Figure 1K). Cox multivariate analysis showed that TNM stages, distant and lymph node metastasis, Lauren classification and FAT4 expression were independent prognostic factors in GC (Table 2).

**Table 2:** Multivariates analysis as determined by Cox regression analysis in 449 GC patients

Clinicopathological parameters	95% Confidential interval		Hazard ratio	*P* value
	Lower	Upper		
Distant metastasis	1.695	1.038	4.447	0.035
TNM stage	1.217	2.133	11.126	0.001
Lauren classification	1.915	1.445	20.393	0.000
Lymph node metastasis	1.591	1.079	5.504	0.019
FAT4 expression	1.043	1.685	5.310	0.021

### *FAT4* mRNA levels are reduced in GC patients with distant metastasis

Next, we analyzed FAT4 mRNA expression in 173 paired GC tissue samples. FAT4 was down-regulated in the tumors than adjacent noncancerous tissues (5.97E-04±1.02E-03 *vs* 9.29E-04±1.21E-03, *P*<0.05, Figure 1L). We classified the patient samples (n=179) based on lymph node (N) and distant metastases (M) status into three groups namely, group1 (N0, M0, n=50), group 2 (N>1, M0, n=64) and group 3 (N>1, M1, n=59). FAT4 expression was higher in group 1 than in group 3 (1.17E-03±0.0013 vs. 2.01E-04±0.0013; Figure 1L, *P*<0.05). FAT4 expression was similar in cancer tissue and adjacent noncancerous tissues in groups 1 (1.17E-03±0.0013 *vs.* 1.44E-03±0.0016, Figure 1L, *P*=0.185) and 2 (5.12E-04±0.00097 *vs.* 7.53E-04±0.00093, Figure 1L, *P*=0.09). FAT4 expression was lower in cancer tissues from group 2 patients than group 1 patients (5.12E-04±0.00097 *vs.* 1.17E-03±0.0013, Figure 1L, *P*<0.05). Moreover, FAT4 expression was lower in cancer tissues than adjacent noncancerous tissues in group 3 patients (2.01E-04±0.00024 *vs.* 6.85E-04±0.0008, Figure 1L, *P*<0.05).

The IHC and qRT-PCR data showed that FAT4 expression was higher at the early stages of the GC progression (N0 or T1+T2 grade), but gradually decreased during lymph node and distant metastatic events (from group1 to group3, Figure 1L). Low FAT4 expression during cancer metastasis was associated with decreased survival (Figure 1K).

### FAT4 promoter methylation is associated with poor prognosis in GC

**Figure 2:**
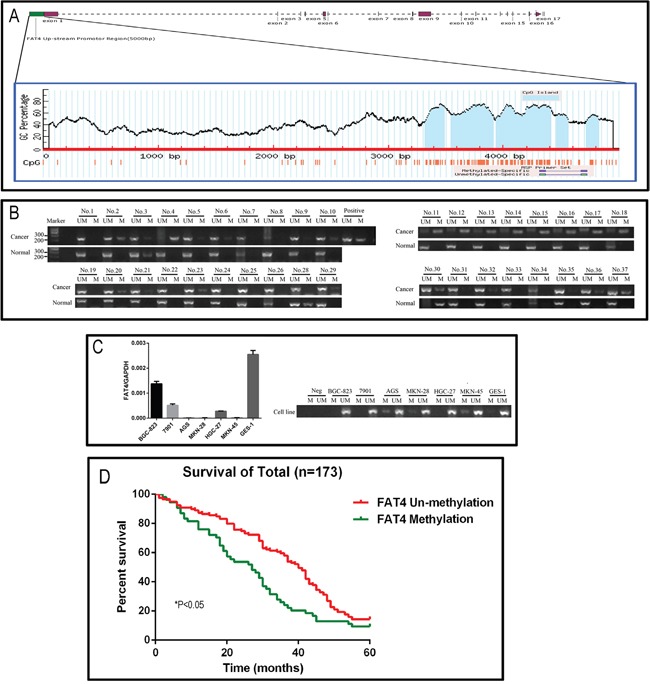
Analysis of FAT4 promoter methylation status and its association with GC **(A)** Schematic representation of the 4.5 kb CpG island region in the human FAT4 gene promoter. **(B)** The representative picture of the Methylation specific PCR analysis of FAT4 promoter region in GC tissues. The samples Nos. 2, 4 and 11-18 show high FAT4 methylation and Nos. 6, 9, 10 and 32 show low methylation. **(C)** FAT4 expression levels and methylation specific PCR analysis of FAT4 promoter region in GC cell lines. **(D)** Kaplan–Meier analysis comparing survival curves of 173 GC patients with and without FAT4 methylation. Patients without FAT4 methylation (red) show increased survival than patients with FAT4 methylation (^*^P<0.05).

Next, we analyzed FAT4 methylation status in 173 paired GC tissue samples to determine if methylation was associated with low FAT4 expression and GC progression. We identified a CpG island region of approximately 4.5 kb in the human FAT4 gene promoter region (Figure 2A). We analyzed the methylation status of the FAT4 promoter region in paired GC and adjacent noncancerous tissues by methylation specific PCR (MSP) analysis. We found that 31.21% (54/173) of GC tissues were methylated (Table 3 and Figure 2B). FAT4 was methylated in AGS, MKN-28 and MKN-45 GC cancer cell lines and unmethylated in the normal GES-1 gastric epithelial cells (Figure 2C). We performed pyrosequencing on 36 GC tissue samples and GC cell lines to verify the results from the MSP method. After re-extraction and quantification, we obtained 32 GC tissues and 6 cell lines for analysis. Four cases (No.7, 23, 29 and 36) had low quality DNA and hence could not be analyzed. We sequenced 6 CpG sites and determined the mean methylation percentage of each site relative to the corresponding matched noncancerous tissue. We classified the specimens based on mean methylation percentages into low (0-5%) and high (> 5%) methylated groups (Supplementary Figure 1). As shown in Supplementary Figure 2B, the pyrosequencing data confirmed the MSP results in high methylation samples (Nos. 2, 4, 11-18; Supplementary Figures 1 and 2) as well as low methylation samples (Nos. 6, 9, 10 and 32; Supplementary Figures 1 and 2). FAT4 promoter showed higher methylation percentage in AGS, MKN-28 and MKN-45 GC cancer cell lines (between 10% and 20%) than 7901, BGC-823, HGC-27 and the normal GES-1 gastric epithelial cells, which showed no methylation (Supplementary Figure 3A). These data demonstrated that FAT4 promoter was methylated in advanced GC cases.

**Table 3:** Association between FAT4 methylation status and clinicopathological factors

Clinical parameters	FAT4		t/χ^2^	*P*
	Unmethylation	Methylation		
Age(yrs)	61.00±13.14	62.50±14.90		
Gender			0.116	0.733
Male	91(69.5%)	40(30.5%)		
Female	28(66.7%)	14(33.3%)		
Location			4.390	0.111
Proximal	12(52.2%)	11(47.8%)		
Middle	45(67.2%)	22(32.8%)		
Distal	62(74.7%)	21(25.3%)		
Size			1.117	0.291
<5cm	61(72.6%)	23(27.4%)		
≥5cm	58(65.2%)	31(34.8%)		
Histology type			6.962	0.073
Papillary adenocarcinoma	1(20.0%)	4(80.0%)		
Tubular adenocarcinoma	96(72.2%)	37(27.8%)		
Mucinous adenocarcinoma	4(57.1%)	3(42.9%)		
Signet-ring cell carcinoma	18(64.3%)	10(35.7%)		
Lauren classification			13.917	0.000
Diffuse type	55(85.9%)	9(14.1%)		
Intestinal type	64(58.7%)	45(41.3%)		
Differentiation			2.413	0.299
Well	0(0.00%)	1(100.0%)		
Moderately	32(66.7%)	16(33.3%)		
Poorly	87(70.2%)	37(29.8%)		
Invasion Depth (T Grade)			11.119	0.011
T1	15(93.8%)	1(6.3%)		
T2	29(74.4%)	10(25.6%)		
T3	64(68.1%)	30(31.9%)		
T4	11(45.8%)	13(54.2%)		
Lymphatic Metastasis (N Grade)			20.846	0.000
N0	43(86.0%)	7(14.0%)		
N1	26(78.8%)	7(21.2%)		
N2	26(68.4%)	12(31.6%)		
N3	24(46.2%)	28(53.8%)		
Distant metastasis (M Grade)			29.093	0.000
M0	94(82.5%)	20(17.5%)		
M1	25(42.4%)	34(57.6%)		
TNM Stages			32.214	0.000
I	26(92.9%)	2(7.1%)		
II	51(85.0%)	9(15.0%)		
III	17(65.4%)	9(34.6%)		
IV	25(42.4%)	34(57.6%)		
Lymphatic metastasis			9.706	0.002
No	43(86.0%)	7(14.0%)		
Yes	76(61.8%)	47(38.2%)		
Vascular invasion			11.978	0.001
No	92(76.7%)	28(23.3%)		
Yes	26(50.0%)	26(50.0%)		
FAT4 expression (IHC Result)			69.677	0.000
Negative	29(36.7%)	50(63.3%)		
Positive	90(95.7%)	4(4.3%)		

All cases were classified according to the World Health Organization's (2010) pathological classification of gastric cancer. Invasion Depth (T Grade) grade T1 includes T1a and T1b, T4 includes T4a and T4b. Lymphatic Metastasis (N Grade) grade N3 includes N3a and N3b. TNM grade I includes Ia and Ib, TNM grade II includes IIa and IIb, TNM grade III includes IIIa, IIIb and IIIc.

### Association between *FAT4* promoter methylation status and GC clinicopathological factors

We treated GC tumor cell lines AGS, MKN-28 and MKN-45 with 1μM of 5-aza-2’-deoxycytidine (5-Aza-dC) for 48h and demonstrated increased expression of FAT4 mRNA (Supplementary Figure 4). This indicated that methylation of the *FAT4* promoter silenced the expression of FAT4.

Nearly 31.2% (54/173) of the GC patients exhibited *FAT4* methylation. Next, we analyzed the association between *FAT4* methylation and expression with the clinicopathological parameters and prognosis of GC. We observed that *FAT4* methylation status was associated with Lauren classification, invasion depth (T Grade), lymphatic metastasis (N grade), distant metastasis (M grade), TNM Stages, vascular invasion and FAT4 expression in GC tissue samples (Table 3). *FAT4* methylation gradually increased from 14.0% to 53.8% in patients with lymphatic metastasis (N0 to N4) and from 7.1% (TNM Grade I) to 34.6% (TNM Grade IV, *P*<0.05). The GC patients with lymph node metastasis exhibited higher *FAT4* methylation than patients without lymph node metastasis (38.2% vs. 14.0%, *P*<0.05). Moreover, patients with distant metastasis demonstrated higher *FAT4* methylation than patients without distant metastasis (57.6% *vs.* 17.5%, *P*<0.05).

### Association between *FAT4* promoter methylation status and GC prognosis

We analyzed the association between *FAT4* methylation status and prognosis of GC patients. In the cohort of 173 patients, the overall survival time was 33.69±1.32 months and the overall survival rate was 12.7%. The median survival time in patients showing *FAT4* methylation was lower than in patients with unmethylated FAT4 (27.00 ± 4.20 months *vs.* 40.00 ±1.72 months, *P*<0.05). The 5-year survival rate of the methylated FAT4 patients was lower than that of the unmethylated FAT4 patients (9.3% vs. 14.3%, *X*^2^*=9.238, P*=0.0024, Figure 2D). Cox multivariate analysis indicated that TNM stage, distant metastasis, lymph node metastasis and vascular invasion were independent prognostic factors (*P*<0.05), but, FAT4 methylation status was not an independent prognostic factor (Hazard Ratio=0.160, *P*=0.689).

### FAT4 levels determine *in vitro* growth and invasiveness of GC cell lines

**Figure 3:**
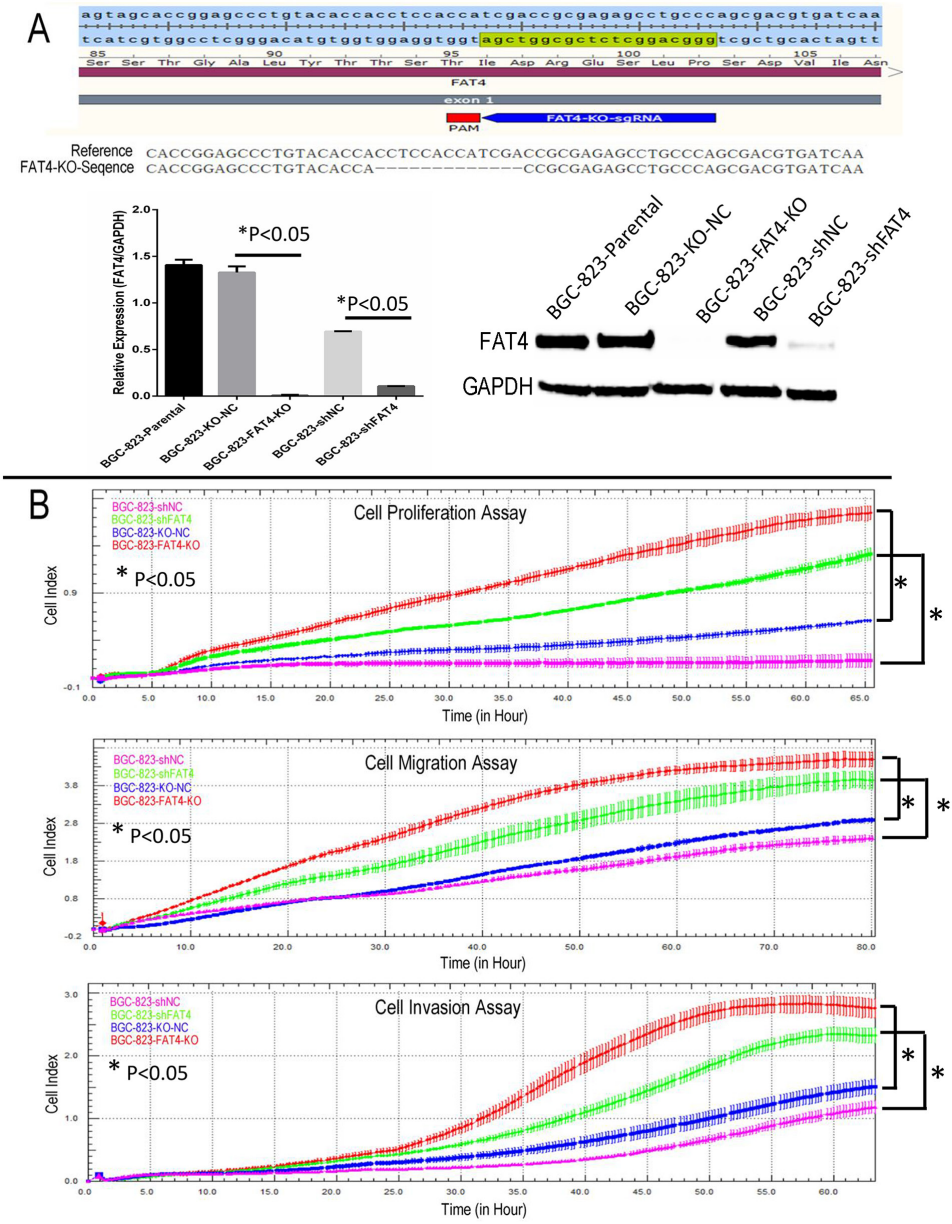
Real time cellular analysis of the effect of FAT4 silencing on GC cell growth, migration and invasion **(A)** Schematic representation of the sgRNA binding site in *FAT4* gene and the *FAT4* knock-out model by CRISPR/CAS9 methodology. **(B)** Real Time Cellular Analysis (RTCA) showing relative cell indices of cellular growth, migration and invasion of BGC-823-FAT4-KO and BGC-823-shFAT4 cells and their corresponding controls, BGC-823-KO-NC and BGC-823-shNC cells. Note:^*^ denotes P<0.05 compared to corresponding controls.

**Figure 4:**
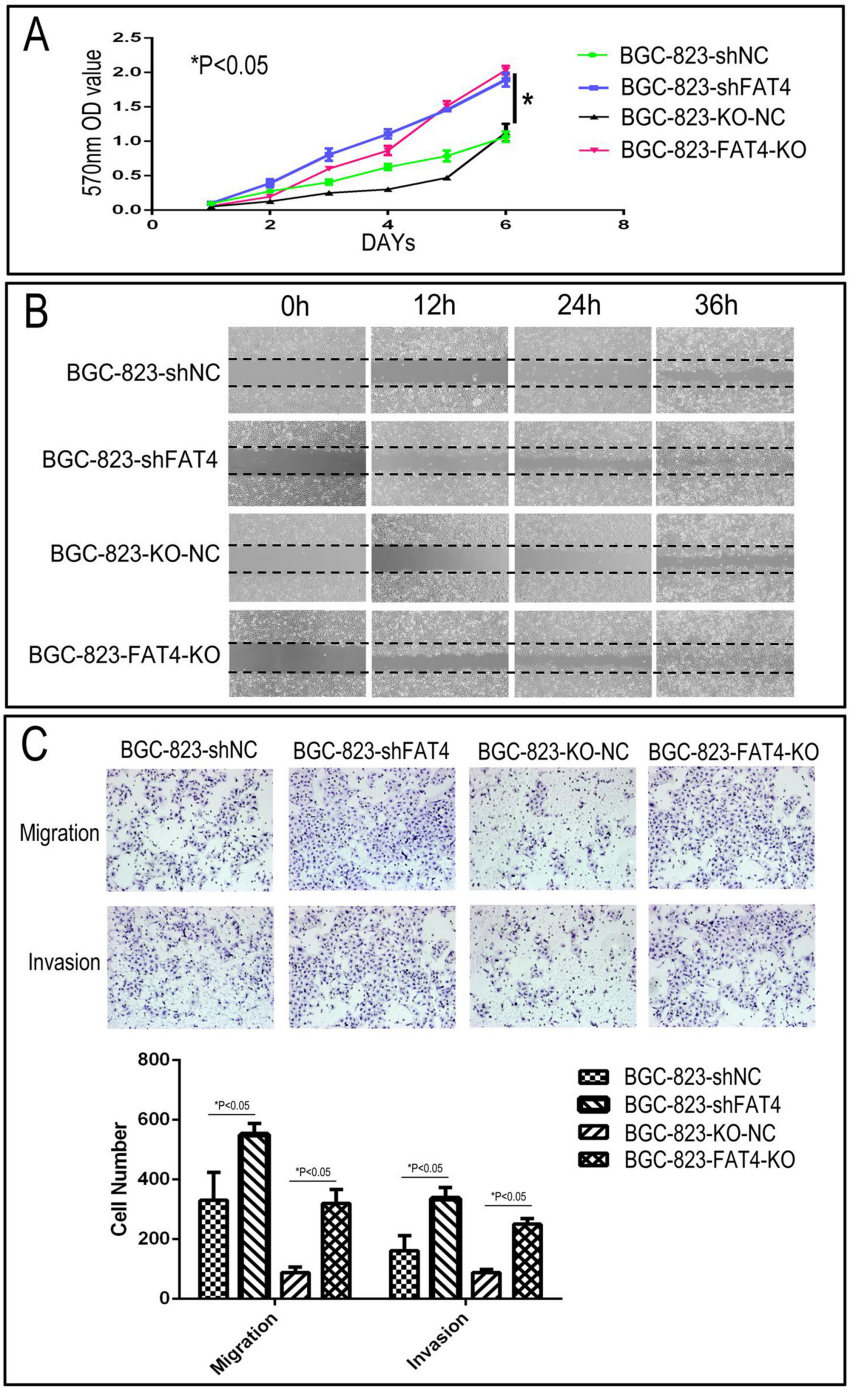
Effect of FAT4 silencing on cell proliferation, migration and invasion of GC cells by MTT, wound healing and Transwell migration and invasion assays **(A)** MTT assay showing cell proliferation rates of BGC-823-FAT4-KO and BGC-823-shFAT4 cells and their corresponding controls, BGC-823-KO-NC and BGC-823-shNC cells. As shown, FAT4 knockdown and knockout cells demonstrate higher proliferation rate than the controls (^*^P<0.05). **(B)** Representative images of wound healing assay showing increased migration of BGC-823-FAT4-KO and BGC-823-shFAT4 cells than their corresponding controls, BGC-823-KO-NC and BGC-823-shNC cells. **(C)** Transwell migration and invasion assays demonstrating increased migration and invasiveness of BGC-823-FAT4-KO and BGC-823-shFAT4 cells than their corresponding controls, BGC-823-KO-NC and BGC-823-shNC cells. Data represent mean ±SD from 3 independent experiments. ^*^ denotes *P* < 0.05 compared to corresponding controls.

We knocked down FAT4 expression in the BGC-823 cell line that exhibits relatively higher FAT4 expression than other GC cell lines (Figure 2C and Supplementary Figure 3B). Western blot and qRT-PCR analysis demonstrated that stable transfection with FAT4-shRNA resulted in decreasing FAT4 expression (Figure 3A). Concurrently, FAT4 expression was knocked down in BGC-823 cells by the sgRNA guided CRISP-CAS9 system (Figure 3A). Analysis by xCELLigence system demonstrated higher cell growth, migration and invasiveness in BGC-823-FAT4-KO and BGC-823-shFAT4 cells than BGC-823-KO-NC and BGC-823-shNC cells (Figure 3B; *P*<0.05). The MTT assay demonstrated that BGC-823-FAT4-KO and BGC-823-shFAT4 cells showed higher proliferation rates than BGC-823-KO-NC and BGC-823-shNC control cells (Figure 4A; *P*<0.05). Wound healing assay showed increased migration of BGC-823-FAT4-KO and BGC-823-shFAT4 cells than the control cells (Figure 4B). Transwell migration and invasion assays showed increased migration and invasiveness of BGC-823-FAT4-KO and BGC-823-shFAT4 cells than the controls (Figure 4C; *P*<0.05). These data suggested that *FAT4* knockdown promoted GC cell proliferation, migration and invasion.

### FAT4 knockout GC cells promote tumorigenesis and lung metastasis in nude mice xenograft model

Next, we tested the effects of FAT4 knockdown on GC tumorigenesis and invasiveness in a nude mice xenograft model by transplanting BGC-823-FAT4-KO and BGC-823-KO-NC cells.

**Figure 5:**
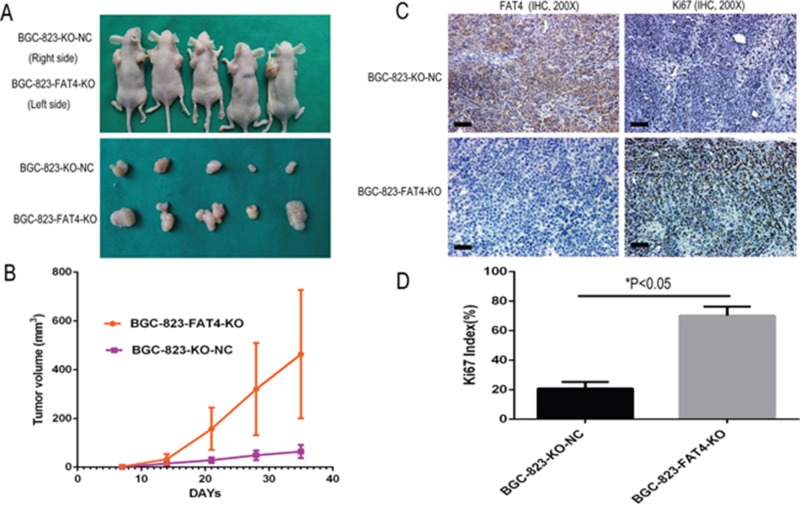
Effect of FAT4 knockout on GC tumorigenesis in the nude mice xenograft model **(A)** Nude mice xenografted (n=5) with BGC-823-FAT4-KO cells (left side) show increased tumor growth (size) than tumors in mice xenografted with control BGC-803-NC cells (right side). **(B)** BGC-823-FAT4-KO cell derived xenograft tumors show increased tumor volume than control BGC-823-KO-NC cells (^*^P<0.05). The data points represent mean tumor volume ± SD measured on the indicated days. **(C)** Representative images show IHC staining of BGC-823-KO-NC and BGC-823-FAT4-KO derived xenograft tumors with antibodies against FAT4 and Ki-67. Scale bar, 100 μm. **(D)** Histogram plots show percent Ki-67 positive cells in BGC-823-KO-NC and BGC-823-FAT4-KO derived xenograft tumors. As shown, the Ki-67 index is higher in the BGC-823-FAT4-KO derived tumors than BGC-823-KO-NC derived tumors. Data represent mean ± SD from 3 independent experiments. ^*^ denotes P < 0.05 compared to control.

In the subcutaneous implant models, we observed increased tumor growth in the BGC-823-FAT4-KO group than the control BGC-803-NC group (Figure 5A). Moreover, BGC-823-FAT4-KO cells showed increased tumor volume than BGC-823-KO-NC cells (463.70 ± 263.58mm^3^ vs. 64.31 ±27.36 mm^3^; Figure 5B, *P*< 0.05).

**Figure 6:**
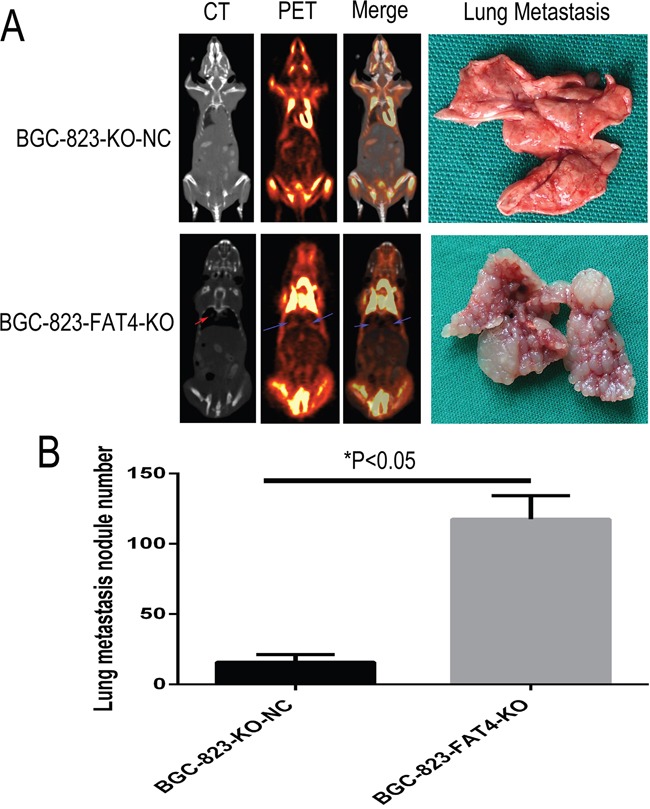
Effect of FAT4 knockout on lung metastasis of GC cells in the nude mice model using PET-CT scans **(A)** PCT-CT scan analysis showing metastasis status at 5 weeks after tail vein injection of BGC-823-FAT4-KO and BGC-823-KO-NC cells into nude mice. CT scan images (left of A) show tumor nodules in the lungs from BGC-823-FAT4-KO group (indicated by red arrow), whereas tumor nodules are absent or less clearly defined in the lungs of the control group. PET scan image (left central of A) and merge analysis image (right central of A) show higher ^18^F-FDG activity in the lungs of BGC-823-FAT4-KO group than the BGC-823-KO-NC group (indicated by blue arrows). The lung metastasis histology figures (top right of A) shown fewer number of lung metastasis nodes in BGC-823-KO-NC, but the numbers of lung metastasis nodes are dramatically increased in BGC-823-FAT4-KO (below right of A). **(B)** Histogram plots show increased number of metastatic tumor nodules in the BGC-823-FAT4-KO group mice (n=5) than BGC-823-KO-NC group (n=5). Data represent mean ± SD from 3 independent experiments. ^*^ denotes *P* < 0.05.

**Figure 7:**
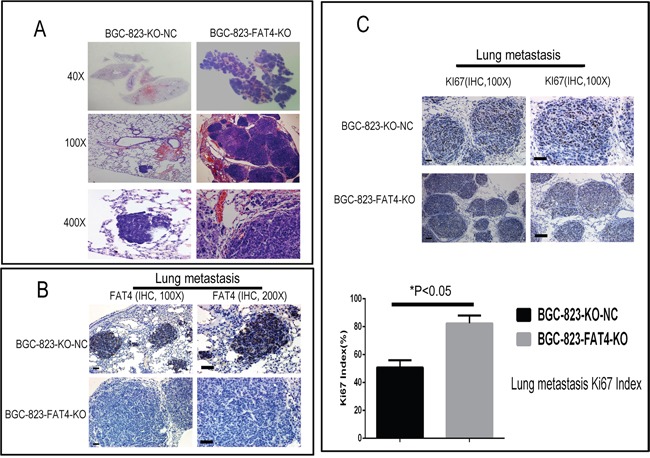
H&E and IHC staining of metastatic lung nodules in the nude mice model **(A)** Representative images (40X, 100X and 400X) of H&E stained lung sections from nude mice injected via the tail vein with BGC-823-FAT4-KO and BGC-823-KO-NC cells. **(B)** Representative images show IHC staining with anti-FAT4 antibody of lung sections from BGC-823-FAT4-KO and BGC-823-KO-NC group mice. FAT4 is positive staining in the lung metastatic tumor nodules of BGC-823-KO-NC group, whereas it negative expresses in BGC-823-FAT4-KO group. Scale bar, 100 μm. **(C)** Histogram shows Ki-67 expression and index (%) in lungs of the BGC-823-FAT4-KO and BGC-823-KO-NC group mice. As shown, the BGC-823-FAT4-KO group shows higher Ki-67 index (%) than BGC-823-KO-NC group. Data represent mean ± SD from 3 independent experiments. Scale bar, 100 μm. ^*^ denotes *P* < 0.05 compared to control.

We analyzed the metastatic status in tail vein injection models after 5 weeks by PET-CT scanning. CT scans indicated the presence of lung nodules in the BGC-823-FAT4-KO group (red arrow), which was absent or ill-defined in the control group (Figure 6A). PET evaluation of the two groups of mice indicated high ^18^F-FDG activity in the brain, bladder, heart and muscle tissues, but not in the lungs. Despite this observation, the BGC-823-FAT4-KO group mice showed higher ^18^F-FDG activity and multiple tumors in the lungs than the BGC-823-KO-NC group mice (Figure 6A; blue arrows). These findings are consistent with the presence of the macroscopic lung metastatic cancer nodules in the lung tissues (Figures 6A and 7A). Gross anatomical analysis of lung metastasis nodules showed higher and larger number of nodules in the BGC-823-FAT4-KO group than the BGC-823-KO-NC group (117.5±16.65 *vs.* 15.83±5.31, *P*<0.05; Figure 6B).

Furthermore, tumors of the BGC-823-FAT4-KO group showed weak FAT4 staining and higher Ki-67 index than the BGC-823-KO-NC group (Figures 5C-5D and 7B-7C). These findings were evident in both subcutaneous implant tumors and lung metastatic cancer nodules. Overall, these results indicate that loss of FAT4 expression promotes GC progression and metastasis.

## DISCUSSION

Many developmental genes that are involved in cell proliferation and apoptosis regulation are deregulated in human diseases including cancers. The *Fat* gene family is a member of the cadherin super family, which controls cell proliferation and planar cell polarity during Drosophila development [13, 14]. In mammals including humans, FAT family members regulate Hippo signaling pathway [9, 12, 13]. Whole-exome sequencing revealed mutations (5%) and deletions (4%) in *FAT4* of GC cases [8]. *FAT4* knockdown increased the migratory and invasive ability of breast cancer cells [9]. The Hippo pathway in mammals involves a kinase cascade that involves phosphorylation and activation of large tumor suppressor 1/2 (LATS1/2) by Ste20-like kinases 1/2 (MST1/2). This kinase cascade inhibits the activities of two transcriptional co-activators, Yes-associated protein (YAP) and transcriptional coactivator with PDZ-binding motif (TAZ) [15]. When YAP and TAZ are activated, they translocate into the nucleus and induce expression of a wide range of genes that regulate tissue homeostasis and organ size control [16]. Dysregulation of the Hippo pathway is associated with cancer development [17]. Mao Y *et al* showed that the planar cell polarity complex was composed of transmembrane cadherins, Fat and Dachsous, which regulated the Hippo pathway [18]. In *Drosophila*, Dachsous binds to Fat, which activates the Hippo pathway by inhibiting the interaction between Zyxin and warts (Wts) (17). In vertebrates, the homolog of Fat is FAT4.

In the present study, loss of FAT4 expression was associated with poor prognosis, tumor size, invasion depth, vascular invasion, lymph node and distant metastases. FAT4 mRNA levels were lower in GC tumor samples than adjacent noncancerous tissues. We observed gradual decrease in FAT4 expression as tumor progressed from a non-metastatic state to tumor associated with lymph node and distant metastasis. FAT4 expression was similar between cancer and adjacent noncancerous tissues in patients without lymph node metastasis. But, FAT4 expression was lower in cancer tissues than adjacent normal tissues in patients with distant metastasis. Our data is in accordance with previous studies that demonstrate the tumor suppressive role of FAT4 in various cancers [9, 19].

In addition to point mutations, epigenetic modifications such as gene methylation inactivate tumor suppressor genes in tumors are very common [20-22]. Methylation of *FAT4* has been reported previously in breast cancer [9]. In the present study, *FAT4* promoter was methylated in 31.2% of GC patients. Methylation of the *FAT4* promoter correlated with FAT4 expression levels. Patients with lymph node and/or distant metastases showed higher methylation at the *FAT4* promoter than patients without metastasis. Therefore, high methylation at the FAT4 gene promoter was associated with poor prognosis for GC patients. The FAT4 promoter region was also methylated to various degrees in different GC cell lines. S-azacytidine, a demethylating agent increased FAT4 expression in GC cell lines. This indicated that the decrease in FAT4 expression was partly due to promoter methylation as previously reported in breast cancer [9] and few stage I lung adenocarcinoma patients [23].

These data suggested that decrease in FAT4 levels was critical for GC progression. The reversal of FAT4 expression alone could not completely block the tumorigenic properties of the GC cells because of acquired genetic changes during their transformation. We demonstrated the critical role of FAT4 in GC progression by showing that FAT4 knockout BGC-823 cells showed increased tumor growth and progression, both *in vitro* and *in vivo*.

In conclusion, our study demonstrates that decreased FAT4 expression is associated with GC progression resulting in poor prognosis. Moreover, *FAT4* promoter methylation decreases FAT4 expression. Thus, our study demonstrates that FAT4 is a potential prognostic marker and therapeutic target for GC patients.

## MATERIALS AND METHODS

### GC patient samples

We performed tissue microarray and immunohistochemical analyses on FFPE samples obtained from 449 GC patients enrolled from January 1998 to January 2004 at the Zhejiang Provincial People's Hospital. The patients were followed up for over 5 years until December 2009. The survival time was estimated from the date of surgery to the end of the follow-up period or the date of death due to carcinoma recurrence. The patients did not receive radiotherapy and/or chemotherapy prior to surgery. We obtained written informed consent from all patients prior to analysis. The age of the GC patients ranged from 17 to 80 (median age: 59.3 y). The patients were classified according to the World Health Organization pathological classification (2010) of tumors. The clinicopathological characteristics of the GC patients are summarized in Table 1. The core tumor area (tumor occupying >50%) was determined by the pathologist based on H&E staining of the 449 FFPE GC tissue wax blocks. Subsequently, 35 (approximately 2 mm diameter) individual paraffin embedded GC blocks were arranged in a recipient paraffin microarray block (tissue array blocks) using a trephine. Finally, 13 tissue array blocks were generated from the 449 GC cases. Each block contained more than three internal controls consisting of normal gastric mucosa.

*FAT4* loss of function was investigated in 173 frozen GC tissue samples. Methylation analysis was conducted on RNA and DNA samples isolated from the 449 samples. This cohort included 50 cases without lymph node and distant metastases, 59 cases with distant metastases (40 with liver metastasis and 19 with ovary metastasis) and 64 cases with lymph node metastasis alone. The clinicopathological characteristics of the GC patients are summarized in Table 3.

### Immunohistochemical staining and evaluation

The GC patient tumor tissue microarray (TMA) and the whole sections of distant metastases tissues were used to analyze FAT4 by IHC. The sections were deparaffinized, rehydrated, and subjected to antigen retrieval combined with signal detection as described previously [24]. The sections were incubated with primary FAT4 antibody (ab198905, 1:100 dilution) overnight at 4°C. They were developed with 3, 3-diaminobenzidine (DAB) and counterstained with hematoxylin. Fat4 staining intensity was determined under a light microscope on a scale of 0–3. The proportion of positively stained cells (0, <5%; 1, 5–25%; 2, 26–50%; 3, 51–75%; 4, 76–100%) were independently estimated by two pathologists in the absence of clinical information as described previously [24, 25]. The intensity and proportion scores were subsequently multiplied to obtain a composite score. A score of 0 to 3 was considered negative, whereas scores of 4 to 12 were considered positive.

### RNA and DNA extraction

The E.Z.N.A. ® DNA/RNA Isolation Kit (R6731-01, Omega Bio-tek, GA, USA) was used to simultaneously isolate genomic DNA and total RNA from 90 GC tissues and cell line extracts. Briefly, the tissue samples were initially lysed and homogenized in a denaturing buffer and the supernatant was applied to a HiBind DNA spin column that binds the DNA. The RNA pellet was dissolved and purified by a HiBind RNA spin column. The purity and concentration of the samples were measured by a NanoDrop 2000 spectrophotometer (Thermo Scientific, USA).

### Quantitative real-time PCR (qRT-PCR)

*FAT4* mRNA levels were analyzed by qRT-PCR. Total RNA from tissue samples and cultured cells was reverse transcribed to cDNA with PrimeScript™ RT Master Mix (Perfect Real Time) kit (RR036A, Takara) according to manufacturer's instructions. Real-time PCR was carried out by the SYBR Green master mix kit in a CFX96 thermal cycling instrument (Bio-Rad). GAPDH was used as endogenous control. The primer sequences were as follows: FAT4-F: 5’-TATCACAAAACGCCCTTGCT-3’, FAT4-R: 5’-TGGATTGTCATTGATATCCTG-3’. GAPDH-F: 5’-TGAAGGTCGGAGTCAACGG-3’ and GAPDH-R: 5’-CTGGAAGATGGTGATGGGATT-3’. The PCR cycling conditions were as follows: 95°C for 4 min followed by 40 cycles of 95°C for 10 s, 60°C for 30 s and 72°C for 30 s. The relative expression was determined by the 2^-ΔΔCt^ method with the Ct values obtained from the melting curves.

### Methylation specific PCR and analysis

The methylation status of *FAT4* gene was determined by methylation specific PCR (MSP) using bisulfite modified genomic DNA as the template. Bisulfite modified genomic DNA was prepared with the EZ DNA Methylation-Gold™ Kit (Zymo Research, D5006, CA, USA) according to the manufacturer's protocol. MSP was carried out in a 50 μl reaction mixture containing 5 μl of bisulfite treated DNA. The MSP assay conditions were as follows: 95°C for 4 min followed by 40 cycles of 95°C for 10 s, 62°C for 30 s and 72°C for 30 s. The reverse primer sequence for both methylated and unmethylated templates was 5’-CCTATATCTAAAATATATAAAAAATC-3’. The forward primers for methylated and unmethylated templates were 5’-GTTTTAGCGGTTATTGTCGGC-3’ and 5’-GTTTTAGTGGTTATTGTTGGT-3’, respectively. After completion of the PCR, the samples were analyzed on a 1.5% agarose gel and the methylated or unmethylated PCR products (∼280 bp) were detected by the Bio-Rad ChemiDoc™ MP System.

### Methylation pyrosequencing

We used pyrosequencing analysis to quantify the methylation levels in 36 pairs of GC and adjacent noncancerous tissues and 7 GC cell lines to verify the MSP results. For bisulfite conversion of the target sequences, EpitTect Bisulfite Kit (Cat No.: 59104, QIAGEN, Germany) was used according to the manufacturer's manual. Pyrosequencing was performed using a PyroMark Gold Q96 Reagents Kit (Cat No.: 972804, QIAGEN, Germany) in a PyroMark Q96 ID System (QIAGEN, Germany) according to the manufacturer's instructions. CpG site methylation was quantified by Pyro Q-CpG 1.0.9 software (QIAGEN, Germany).

In brief, double stranded PCR products were denatured with 0.1M sodium hydroxide (NaOH) followed by PBS washes. It was then annealed to the sequencing primer (5’-CTACAACCCCCCTCCA-3’). The pyrosequencing reaction was started at the 3’-end of the sequencing primer by adding A, T, C, and G nucleotides into each sample well, one at a time. As the bases were incorporated into the growing DNA strand based on complimentary base pairing, each incorporated base resulted in light production as a result of activation of an enzymatic cascade. The light intensity was graphed in a program and analyzed by the Pyromark CpG software to determine average methylation at all CpG sites.

### *FAT4* knock-out by the CRISPR/Cas9 system

To knockout FAT4, sgRNA sequence, 5’- GGGCAGGCTCTCGCGGTCGA-3’ that targeted exon1 of FAT4 was designed with the Zhang Lab web application (http://crispr.mit.edu/). The FAT4 specific sgRNA was cloned into the pL6-U6-Bbsl_Bbsl_gRNA scaffold plasmid (Addgene, Cambridge, USA), linearized by digestion with Bbsl (Thermo Scientific, ER1011, USA) for 30 min at 37°C and purified by gel electrophoresis. Subsequently, pair of oligonucleotide sequences was designed to target the FAT4 exon 1, namely, FAT4-KO-sgRNA-F: ACCGGGGCAGGCTCTCGCGGTCGA and FAT4-KO-sgRNA-R: AAACTCGACCGCGAGAGCCTGCCC. The two oligonucleotides were annealed, phosphorylated and ligated to the linearized pL6 vector. BGC-823 GC cells were transfected in a 6-well plate with 250 ng of pL6-U6-Bbsl_Bbsl_gRNA scaffold and/or an empty pL6-U6-Bbsl_Bbsl_gRNA scaffold plasmid and 500 ng of pXC-FLAG-hCas9 plasmid using polyethylenimine (PEI, Polyplus transfection^®^ SA, 115-010, USA). After 48 h, the cells were selected for 3 weeks with 1 μg/ml puromycin. Then, single colonies were tested for authentic clones by PCR amplification of the CRISPR binding site using the following primers: forward: 5’-CCCGTTGCACACTCTATCAGTATCT-3’ and reverse: 5’-TGTCTTGCACAGTCACGTTTACCT-3’. PCR products were analyzed by Sanger sequencing.

### *FAT4* shRNA gene silencing

The FAT4-shRNA hairpin DNA sequences (forward: 5’-CACCGCGCATTGTTAGATAGGGAAACTCGAGTTTCCCTATCTA CAATGCGCTTTTTTG-3’; reverse: 5’-AGCTCAAAAAAGCGCATTGTTAGATAGGGAAACTCG AGTTTCCCTATCTAACAATGCGC-3’) were annealed and cloned into pYr-1.1 vector (Yinrun Biotechnology, Changsha, China). The plasmid was linearized by BsaI restriction enzyme and transfected into BGC-823 cells with lipofectamine 3000 (Invitrogen, USA) according to manufacturer's instructions. Stable cell lines were selected with 200 mg/ml G418 treatment for 3 to 4 weeks (BGC-823-shFAT4). The control cells were generated by transfecting BGC-823 cells with pYr-1.1 vector with nonsense shRNA (BGC-823-shNC).

### Real time cellular analysis (RTCA) of cellular growth, migration and invasion

The xCELLigence system (ACEA Biosciences Inc., Hangzhou, China) was used in order to monitor the cellular growth. The system consisted of microtiter plates (E-plates) with integrated gold microarrays at the bottom of the wells for continuous real time and label-free measurements of cellular status by electrical impedance. Cell index (CI) was determined based on relative changes in impedance at different time points (T) in comparison to baseline (T_0_). In standard E-plates, the CI values are proportional to the number of cells attached. The kinetic profiles reflect adhesion and spreading in approximately 6 h following cell seeding. In the present study, BGC-823-FAT4-KO, BGC-823-shFAT4 and the corresponding control cells were seeded in E-plates at a density of 5000 cells/well in 10% FBS supplemented RPMI-1640 medium. The E-plates were transferred to the RTCA-DP instrument for automated real-time monitoring at standard incubator conditions with quadruplet readouts of CI every 30 min for the following 3 days.

The rates of migration and invasion were further monitored by the xCELLigence-system in cell invasion or migration (CIM) plates as previously described [26]. CIM plates have microelectronic sensors located on the underside of a microporous membrane insert. The migrating cells adhere to the sensors resulting in higher impedance and CI. For the migration assays, BGC-823-FAT4-KO, BGC-823-shFAT4 and the corresponding control cells were seeded in the upper chamber of the CIM-plates in serum-free RPMI 1640 medium at a density of 40000 and 200000 cells per well for migration and invasion assays, respectively. The bottom chambers were filled with 30% FBS supplemented RPMI 1640 medium to promote migration across membranes by generating a serum gradient. For the invasion assays, the upper chambers were loaded with 30 μl of 1:20 diluted matrigel to generate a 3D biomatrix film. Following cell seeding, CIM-plates were transferred to the RTCA instrument for continuous readouts during the next 70 h. CI was registered solely by cells that migrated through the 8 μm porous membranes. The data were analyzed by the RTCA software 2.0.

### MTT, wound healing and transwell assays

The RTCA data of cell growth, wound healing, migration and invasion were confirmed by the MTT, wound healing and the Transwell assays as previously described [25].

### Western blot analysis

Equal amounts of total tissue or cell protein samples were subjected to 10% SDS-PAGE at 150V for running 1h and then wet transferred overnight onto 0.45μm PVDF membranes. The membranes were blocked with 5% nonfat dry milk in 1X PBST buffer. Then, the blots were probed with primary antibodies FAT4 (ab130076, 1:1000, Cambridge, MA, USA) and GAPDH (ab128915, 1:3000, Cambridge, MA, USA) followed by incubation with the corresponding HRP-conjugated secondary antibodies (ab205718, 1:5000, Cambridge, MA, USA). The blots were developed with enhanced chemiluminescence (ECL) reagent (#34077, Thermo Fisher, USA) and imaged in the Bio-Rad ChemiDoc™ MP system(Bio-Rad Laboratories, Inc.USA).

### Nude mice xenografts

Four to Five week old female BALB/c athymic nude mice were purchased from Slac Laboratory Animal Co. Ltd. (Shanghai, China). They were housed in pathogen-free conditions according to our institutional guidelines. The study protocol was approved by the Animal Care committee of our institution.

The mice (n=5) were simultaneously injected with BGC-823-FAT4-KO and BGC-823-KO-NC cells (2 X 10^6^ cells/100 μl, in the left and right flanks, respectively. Tumor size was measured every 7 days by a slide caliper and tumor volume was calculated as (length×width^2^)/2.

To evaluate the effect of FAT4 on GC metastasis, 1×10^6^ BGC-823-FAT4-KO and BGC-823-KO-NC cells were injected into the lateral tail vein of the mice. Metastatic lung nodules were scanned by whole-body micro-PET/CT and quantified by H&E staining using a dissecting microscope at each endpoint. The mice were sacrificed after 30 days and subcutaneous tumor or metastatic lung tissues were rapidly collected. A portion of the tissue was stored in liquid nitrogen, whereas the remaining was fixed in 10% neutral buffered formalin and embedded in paraffin. The tissues were cut into 4 μm thick sections and stained with H&E. Some sections were subjected to IHC staining with antibodies against FAT4(NBP1-78381F, rabbit polyclonal anti-FAT4 antibody, 1: 150, Novus Biologicals, Littleton, CO, USA) and Ki-67 (#GA62661, Clone MIB-1, Ready to use, DAKO, USA). The Ki-67 proliferation index was determined by a senior pathologist by estimating the percentage of positive nuclei in 1,000 cells in hot spots.

### Whole-body ^18^F-FDG PET/CT

Nude mice with GC xenografts were injected with approximately 0.2 mCi of ^18^F-FDG for 1 h under continuous anesthesia. The mice were placed at the centre of the Siemens Inveon combined microPET-CT scanner (Siemens Preclinical Solution USA, Inc., Knoxville, TN, USA) in a prone position. MicroCT scans were conducted under the following conditions: X-ray tube voltage: 80 kV; current intensity: 500 μA; exposure time: 150 ms and 120 rotation steps. PET static acquisition was carried out for 10 min and the images were reconstructed using the OSEM (ordered set expectation maximization) algorithm for 3D PET reconstruction. The images were analyzed by the Inveon Research Workplace 4.1 (Siemens, Erlangen, Germany). The standardized uptake (SUV g/ml) was determined as SUV = [(RTA/cm^3^)/RID] × BW (RTA is the measured radiotracer tissue activity in mCi; RID is the radiotracer injected dose in mCi and BW is the mouse body weight in grams).

### Statistical analysis

All statistical analyses were performed with the SPSS 13.0 statistical software. Differences between groups were estimated by 2-tailed paired Student's t test or independent sample t-test. The relationship between FAT4 expression and clinicopathological characteristics were analyzed by the Chi-square test. Kaplan-Meier survival curves were analyzed by the log-rank test. The significance of various survival related variables were assessed by Cox regression model in the multivariate analysis. *P*<0.05 was considered statistically significant.

## SUPPLEMENTARY MATERIALS FIGURES


