# Supplementary material for: Low FAT4 expression is associated with a poor prognosis in gastric cancer patients
Source: Oncotarget. 2017 Dec 26;9(4):5137–54. doi: 10.18632/oncotarget.23702 (PMC5797039; doi:10.18632/oncotarget.23702)
Supplement: Supplementary file 1 [file oncotarget-09-5137-s001.pdf]

Low FAT4 expression is associated with a poor prognosis in gastric cancer patients

SUPPLEMENTARY MATERIALS

Methylation Rate (%)							Sample No.	Meth. Satus
Pos. 1	Pos. 2	Pos. 3	Pos. 4	Pos. 5	Pos. 6	Ave.		
Pos. 1 Meth. (%)	Pos. 2 Meth. (%)	Pos. 3 Meth. (%)	Pos. 4 Meth. (%)	Pos. 5 Meth. (%)	Pos. 6 Meth. (%)	Mean Meth. (%)		
						39.89	No.4	High Methylated (>5%)
						34.52	No.12	
						20.1	No.18	
						19.54	No.14	
						18.13	No.15	
						14.2	No.11	
						13.96	No.13	
						13.19	No.16	
						11.07	No.17	
						8.745	No.2	
						6.525	No.37	
						6.315	No.20	
						4.783	No.10	Low Methylated (<5%)
						3.493	No.32	
						2.462	No.9	
						0.258	No.6	
						-1.81	No.21	Un-Methylated
						-2.69	No.31	
						-4.29	No.5	
						-4.79	No.19	
						-5.42	No.22	
						-5.83	No.24	
						-7.78	No.25	
						-8.66	No.26	
						-10.8	No.1	
						-11	No.33	
						-11.5	No.35	
						-11.9	No.8	
						-12.2	No.34	
						-12.5	No.3	
						-13.3	No.30	
						-13.3	No.28	

Supplementary Figure 1: Methylation pyrosequencing analysis of *FAT4* promoter in GC tissue samples. Histogram shows mean methylation percentage based on pyrosequencing of 6 CpG sites in the FAT4 promoter region of GC tumor tissues relative to adjacent noncancerous tissues (n=32). Note: if mean methylation percentage was lower than 0%, the samples are referred to as unmethylated; if mean methylation percentage was between 0-5% or >5%, they are referred to as low and high methylated. Both low- and high- methylated groups were included in methylated group.

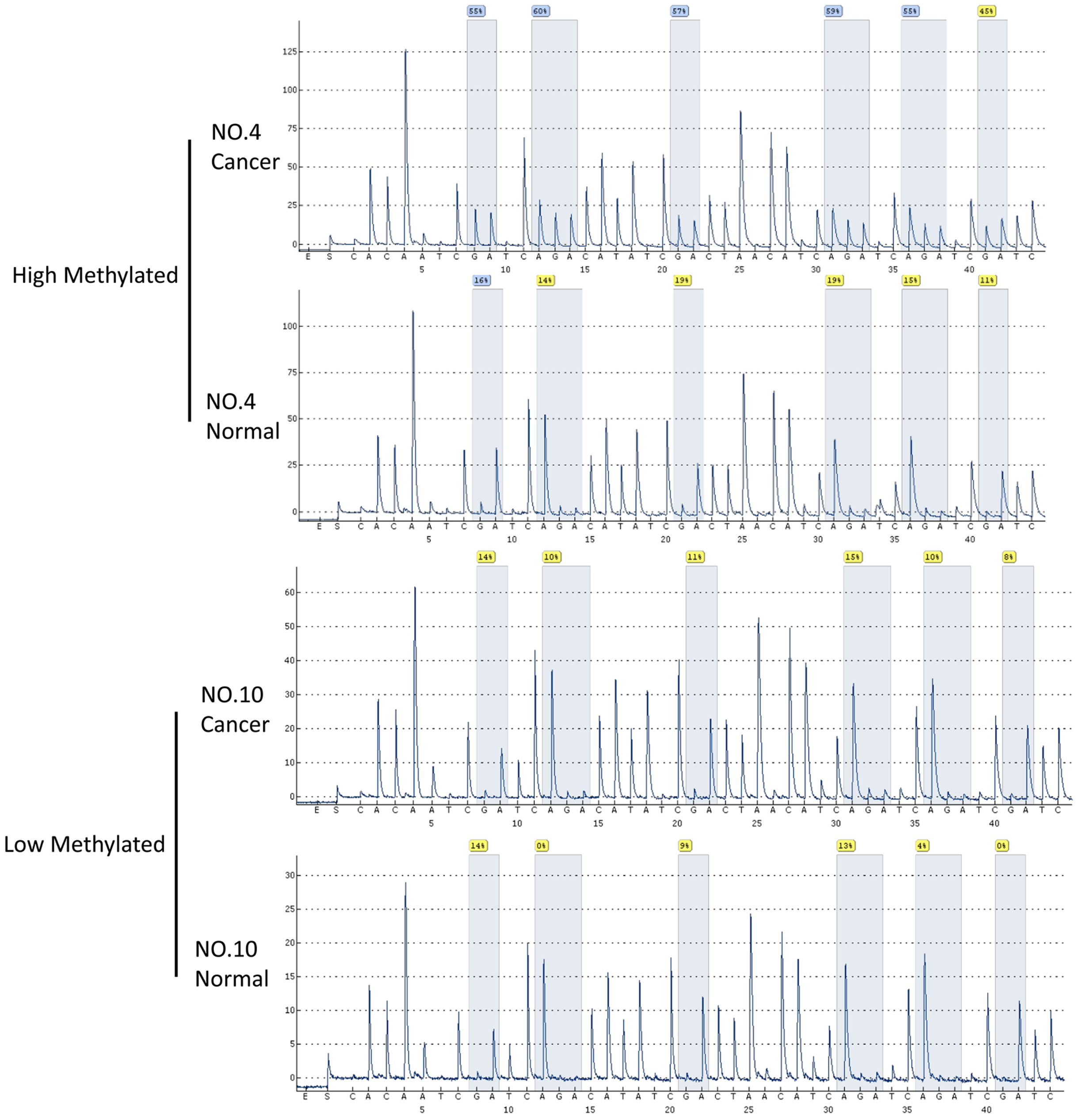

Supplementary Figure 2: Representative pyrosequencing results showing methylation patterns in high and low methylated GC patient samples.

A

Methylation Percentage (%)							
Pos. 1 Meth. (%)	Pos. 2 Meth. (%)	Pos. 3 Meth. (%)	Pos. 4 Meth. (%)	Pos. 5 Meth. (%)	Pos. 6 Meth. (%)	Mean Meth. (%)	Samples
0.05	0.02	0.03	0.05	0.03	0.10	**0.05**	BGC-823
0.08	0.06	0.09	0.09	0.06	0.05	**0.07**	7901
20.36	26.53	21.71	23.08	16.41	14.93	**20.50**	AGS
10.69	10.11	11.16	10.40	12.51	11.00	**10.98**	MKN-28
0.08	0.06	0.09	0.09	0.06	0.05	**0.07**	HGC-27
15.99	12.51	15.22	20.01	21.37	10.44	**15.92**	MKN-45
0.06	0.05	0.10	0.04	0.05	0.05	**0.06**	GES-1

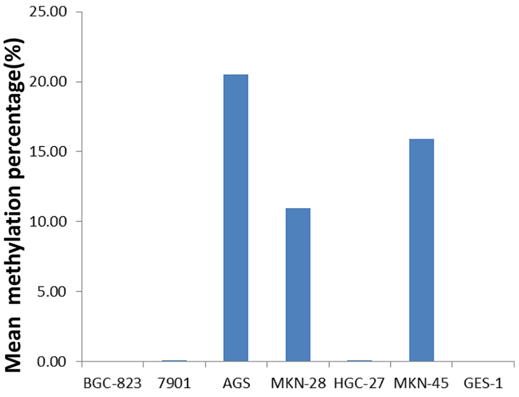

B

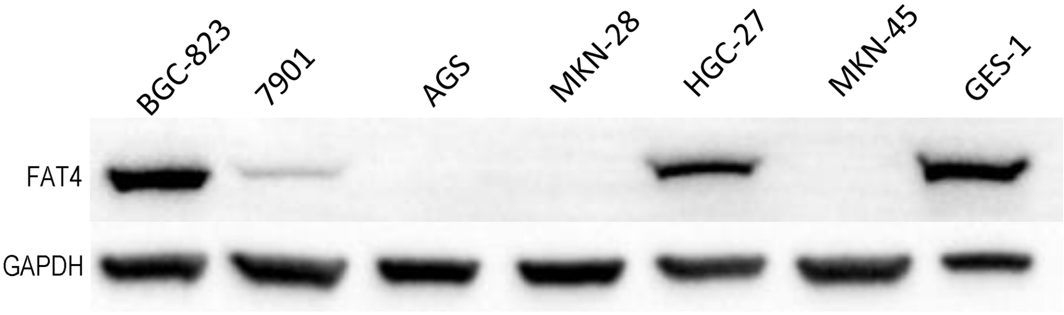

Supplementary Figure 3: FAT4 promoter methylation pyrosequencing of GC cell lines and FAT4 expression in GC cell lines. **(A)** Mean methylation percentages of the *FAT4* promoter in various GC cell lines. The AGS, MKN-28 and MKN-45 cells show *FAT4* promoter methylation, the mean methylation percentage of between 10% and 20%. However, the GES-1, HGC-27, 7901 and BGC-823 cells shown no *FAT4* promoter methylation. **(B)** Representative western blots demonstrating FAT4 expression in various GC cell lines. The GC cells BGC-823, GES-1, 7901 and HGC-27 shown FAT4 expression, whereas AGS, MKN-28 and MKN-45 shown no FAT4 expression. GAPDH was used as loading control.

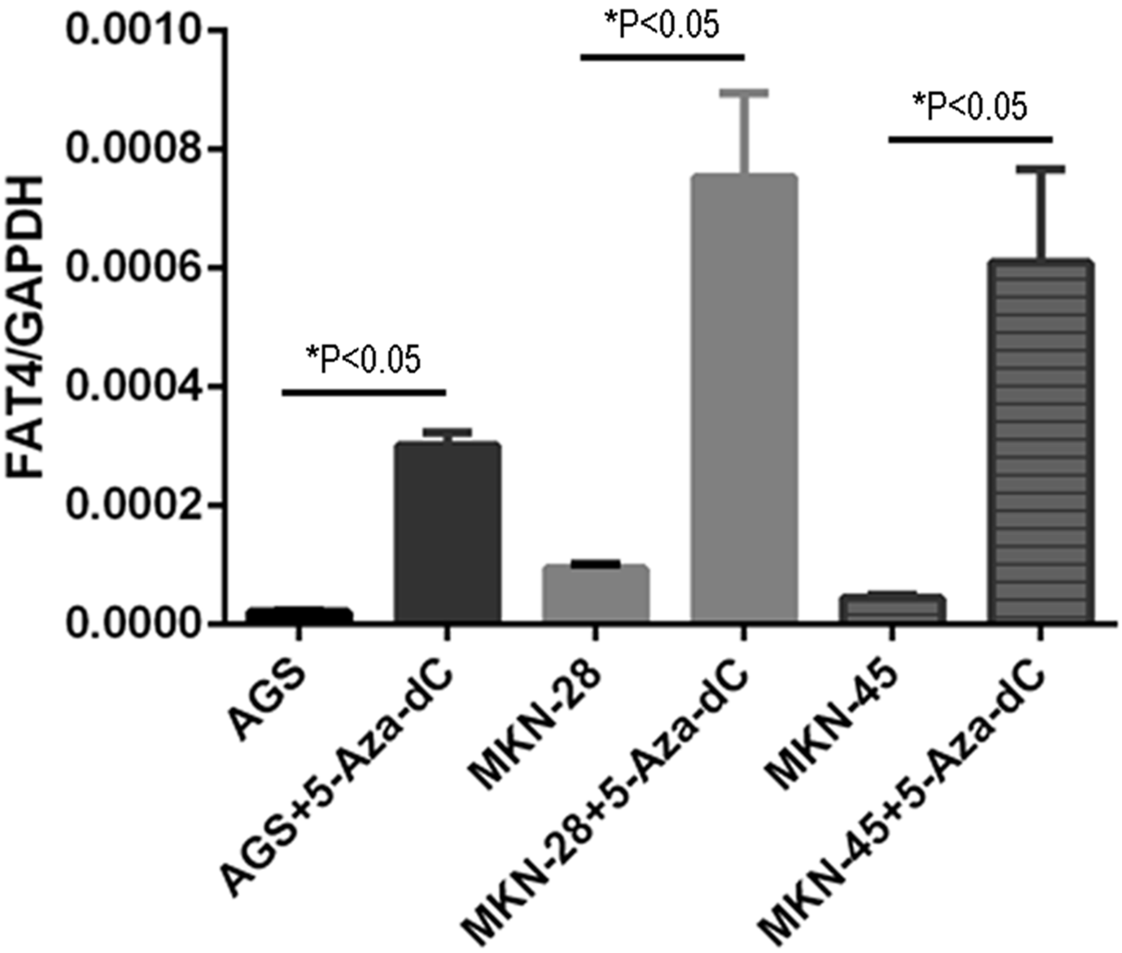

Supplementary Figure 4: 5-aza deoxycytidine increases FAT4 expression in BGC-823 cells. qRT-PCR analysis showingFAT4 levels in untreated and 5-Aza-Dc (1μM) treated BGC-823 GC cells. As shown, FAT4 levels increased upon 5-Aza-dC treatment (*P<0.05).
